# THE POWER OF REVIEWS: HARNESSING RESEARCH ON COGNITIVE HEALTH

**DOI:** 10.1093/geroni/igz038.2212

**Published:** 2019-11-08

**Authors:** Kelly Quinn, Daniela B Friedman

**Affiliations:** 1 University of Illinois at Chicago, Chicago, Illinois, United States; 2 University of South Carolina, Columbia, South Carolina, United States

## Abstract

Networks harness powerful resources to tackle complex challenges. This symposium leverages the power of one such network to advance research in cognitive health. Using literature reviews, we seek to aid in the translation of knowledge on cognitive aging and health into practice. By documenting research successes and identifying gaps, we highlight areas in which important lines of research can be propelled forward. The papers together focus on three dynamics of research practice: communication among researchers, commonly-used research tools, and the need to develop valid and reliable measures. The first paper examines nomenclature that scientists use to describe cognitive health in their research reporting, and makes recommendations for alignment of important concepts. The second and third papers explore commonly-used tools in intervention research: one on the use of psychosocial assessments typically employed with individuals with ADRD, such as for depression and quality of life, and the other on tools used by educational and intervention programs to evaluate caregiver knowledge of dementia. Each paper suggests that consistent measurement practices would enhance interpretation of findings across studies. The final paper considers a promising research area, walkable neighborhoods and cognitive health, and argues for the importance of developing valid and reliable measures in emerging research. Taken together, these papers provide critical reflection on current practices in cognitive aging research and suggest directions in which to build future investigation. In addition, this collection of papers fulfills the clarion call of The Healthy Brain Roadmap for finding ways to improve the cognitive health of all adults.

